# A Single Injection of rAAV-shmTOR in Peripheral Nerve Persistently Attenuates Nerve Injury-Induced Mechanical Allodynia

**DOI:** 10.3390/ijms242115918

**Published:** 2023-11-02

**Authors:** Minkyung Park, Ha-Na Woo, Chin Su Koh, Heesue Chang, Ji Hyun Kim, Keerang Park, Jin Woo Chang, Heuiran Lee, Hyun Ho Jung

**Affiliations:** 1 Department of Neurosurgery, Yonsei University College of Medicine, Seoul 03722, Republic of Korea; mkpark3142@yuhs.ac (M.P.); cskoh@yuhs.ac (C.S.K.); hsjang13@yuhs.ac (H.C.); jchang@yuhs.ac (J.W.C.); 2 Brain Korea 21 PLUS Project for Medical Science and Brain Research Institute, Yonsei University College of Medicine, Seoul 03722, Republic of Korea; 3 Department of Biochemistry & Molecular Biology, University of Ulsan College of Medicine, Seoul 05505, Republic of Korea; woohn101@gmail.com; 4 Bio-Medical Institute of Technology, University of Ulsan College of Medicine, Seoul 05505, Republic of Korea; jihyun.kim1110@gmail.com; 5 Department of Microbiology, Asan Medical Center, College of Medicine, University of Ulsan, Seoul 05505, Republic of Korea; 6 Cedmogen Co., Ltd., Cheongju 28644, Republic of Korea; cdmogen@cdmogen.com

**Keywords:** neuropathic pain, mammalian target of rapamycin (mTOR), recombinant adeno-associated virus (rAAV), dorsal root ganglion (DRG)

## Abstract

Activation of mammalian target of rapamycin (mTOR) has been known as one of the contributing factors in nociceptive sensitization after peripheral injury. Its activation followed by the phosphorylation of downstream effectors causes hyperexcitability of primary sensory neurons in the dorsal root ganglion. We investigated whether a single injection of rAAV-shmTOR would effectively downregulate both complexes of mTOR in the long-term and glial activation as well. Male SD rats were categorized into shmTOR (*n* = 29), shCON (*n* = 23), SNI (*n* = 13), and Normal (*n* = 8) groups. Treatment groups were injected with rAAV-shmTOR or rAAV-shCON, respectively. DRG tissues and sciatic nerve were harvested for Western blot and immunohistochemical analyses. Peripheral sensitization was gradually attenuated in the shmTOR group, and it reached a peak on PID 21. Western blot analysis showed that both p-mTORC1 and p-mTORC2 were downregulated in the DRG compared to shCON and SNI groups. We also found decreased expression of phosphorylated p38 and microglial activation in the DRG. We first attempted a therapeutic strategy for neuropathic pain with a low dose of AAV injection by interfering with the mTOR signaling pathway, suggesting its potential application in pain treatment.

## 1. Introduction

Injuries to the peripheral nervous system result in severe neuropathic pain, which is characterized by the dysfunction of the somatosensory system [1]. It affects the normal perception of pain, such as with mechanical allodynia, an abnormal hypersensitivity to innocuous stimuli. The primary sensory neurons within the dorsal root ganglion (DRG) are mainly responsible for transmitting peripheral sensory signals and pain perception to the central nervous system [2]. In particular, because of its incomplete blood–nerve barrier, its permeability is higher than that of the central nervous system, allowing more neurotoxins to easily penetrate the DRG and increasing the likelihood of neuronal excitability after injury [3]. Given that the therapeutic region can be focal and restricted, as opposed to supraspinal, it is noteworthy to consider neuroplastic changes in the DRG in relation to chronic pain [4]. Following an injury, a large population of glial cells surrounding the primary sensory neurons releases inflammatory cells to protect neurons and regulate immune responses in the DRG [5]. Activated glial cells proliferate and alter their phenotypes, followed by chemicals released from the injury site, resulting in neuronal sensitization by prolonged stimulation of the neurons [6]. Hence, increasing evidence suggests that glial activation and glial-driven inflammatory mediators are largely implicated in the maintenance of pain, and are regarded as critical factors to consider [7].

Mammalian target of rapamycin (mTOR), a multiprotein complex composed of mTOR Complex 1 (mTORC1) and mTOR Complex 2 (mTORC2), is a serine/threonine protein kinase that is a master regulator of cell growth and proliferation, protein translation, and autophagy [8]. Numerous diseases, including ocular disorders, cancer, diabetes, and neurodegenerative diseases, have been linked to the activation of the mTOR signaling pathway [9,10]. Its activation leads to the phosphorylation of downstream effectors such as p70 ribosomal S6 protein kinase (p70S6K1) and 4E-binding protein 1 (4E-BP1) and further promotes protein synthesis in cell bodies, axons, and dendrites [11]. Several studies have identified an increased level of phosphorylated mTOR as well as downstream effectors in various types of neuropathic pain models, which were reversed by the systematic administration of an intrathecal or intracerebral injection of rapamycin [12,13,14,15,16]. Based on previous reports, it is evident that mTOR and its downstream effectors are activated under chronic pain conditions [11]. However, it has been demonstrated that rapamycin or rapalogs, two widely used mTOR inhibitors, selectively downregulate mTORC1. It has been suggested that the inhibition of mTORC1 alone may trigger a negative feedback loop in the mTORC1 pathway, which in turn activates Akt signaling. Once Akt activation is elevated, it results in the long-term survival of certain cell types, resulting in an incomplete suppression of mTOR and pain hypersensitivity [17,18]. To overcome the limitations of first-generation inhibitors, second-generation inhibitors and ATP-competitive inhibitors, including Torin1 and pp242, have been recently developed, but only a few studies have been conducted using the acute inflammatory pain model. Recent studies have revealed that mTOR inhibitors, both first- and second-generation inhibitors, are somewhat effective in animal models of neuropathic pain. However, most of them showed only short-term effects, and the animal models used in these studies tended to focus on acute pain models [19,20,21].

To address current issues, we designed a short hairpin RNA (shRNA) packaged in a recombinant adeno-associated virus (rAAV) that induces a long-lasting transgene expression by selectively inhibiting mTORC1 and mTORC2 activities. It has been demonstrated that rAAV has no pathogenicity, a low host immune response, and the ability to infect both dividing and non-dividing cells. The preclinical and clinical applications of rAAV vectors have already been characterized as one of the most promising tools for inducing stable, long-term effects without repetitive treatments. To date, the underlying mechanism of mTOR in neuropathic pain remains unknown because of discrepancies in the etiologies of neuropathic pain models and different systemic administrations. To better understand the role of the mTOR signaling pathway in chronic pain mechanisms, we hypothesized that a low dose of rAAV-shmTOR would prolong the treatment effects in mechanical allodynia on a long-term basis and effectively downregulate both complexes of mTOR and glial activation.

## 2. Results

### 2.1. Long-Term Changes in Mechanical Hypersensitivity with rAAV-shmTOR Treatment

**Figure 1:**
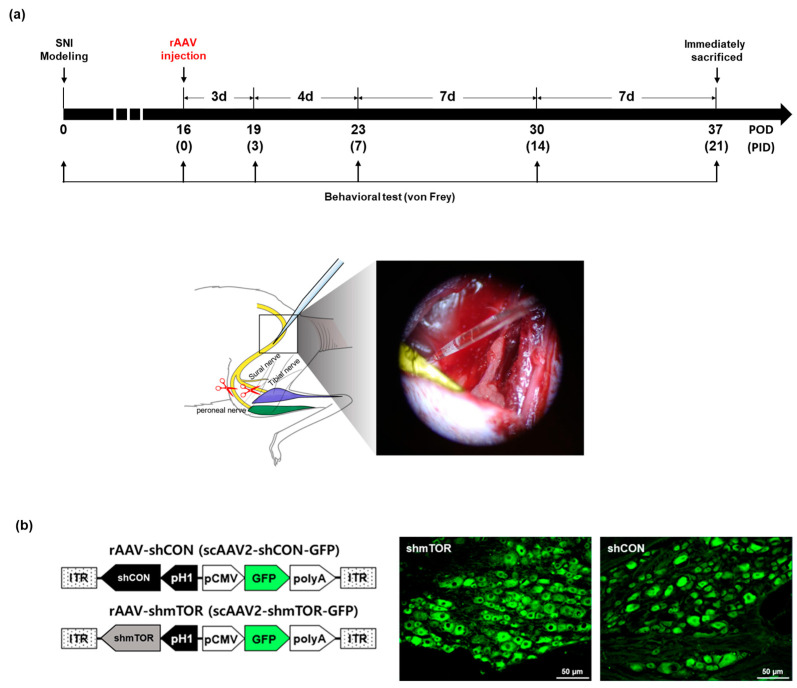
An illustration of the experimental scheme. Scale bar = 50 µm. (**a**) The experimental timeline and schematic diagram of the rAAV injection system. (**b**) The schematic representation of the rAAVs used in this study and the GFP expression in the DRG on PID 21 in the shmTOR group (**left**) and shCON group (**right**).

**Figure 2:**
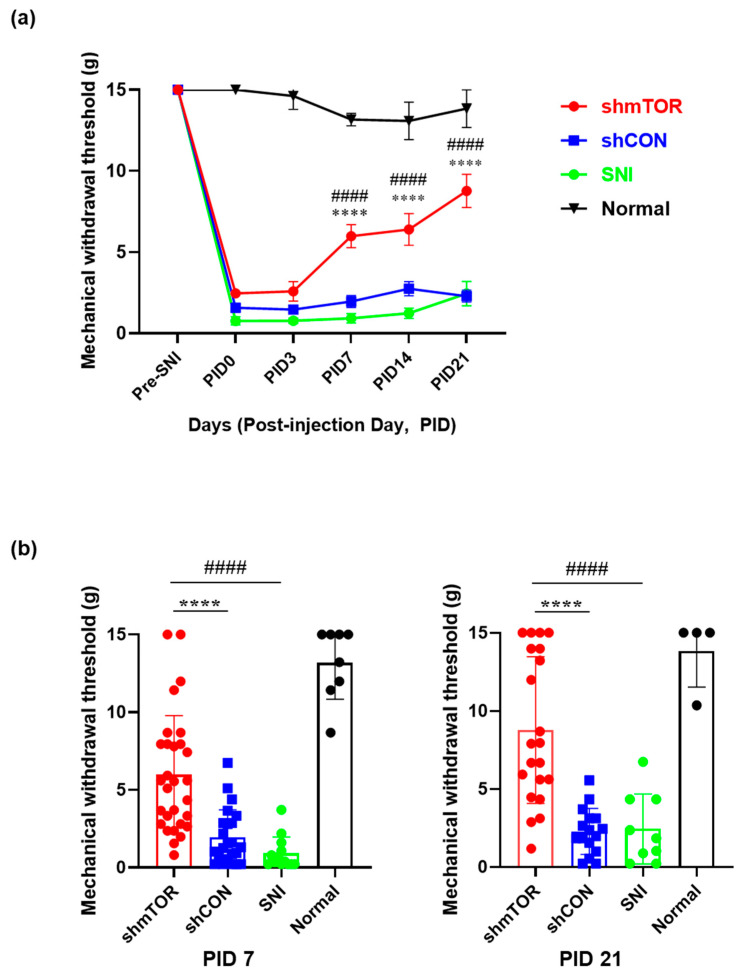
Long-term behavioral test for mechanical allodynia. (**a**) Changes in long-term mechanical withdrawal threshold following the rAAV injection. The mechanical withdrawal threshold in the shmTOR group gradually increased while that of the shCON group consistently remained low throughout the experiment (**** shmTOR vs. shCON, #### shmTOR vs. SNI, data are expressed as mean ± SEM, **** *p* < 0.0001, #### *p* < 0.0001, two-way ANOVA with Tukey’s multiple comparisons test (group, F(3, 368) = 208.7, *p* < 0.0001; PID, F(5, 368) = 163.2, *p* < 0.0001; group and PID interaction, F(15, 368) = 15.59, *p* < 0.0001). (**b**) Mechanical withdrawal threshold comparison at two different time points, PID 7 and PID 21. One-way ANOVA with Tukey’s multiple comparisons test. (**** shmTOR vs. shCON, #### shmTOR vs. SNI, data are expressed as mean ± SEM. PID 7, shmTOR vs. shCON, **** *p* < 0.0001; shmTOR vs. SNI, #### *p* < 0.0001; PID 21, shmTOR vs. shCON, **** *p* < 0.0001; shmTOR vs. SNI, #### *p* < 0.0001).

Sixteen days after spared nerve injury (SNI) surgery, rAAV was injected in the sciatic nerve and behavioral tests were conducted on post-injection day (PID) 3, 7, 14, and 21 to investigate long-term effects (Figure 1a). In order to confirm that the virus was correctly delivered, we investigated the GFP expression in the DRG on PID 21. It was demonstrated that GFP was predominantly expressed in the DRG up to PID21 (Figure 1b). Long-term behavioral test showed that injury induced persistent mechanical allodynia for 5 weeks, which is not shown in the uninjured group. On PID3, the shmTOR, shCON, and SNI groups were significantly different only from the Normal group (shmTOR: 2.585 ± 0.600, shCON: 1.461 ± 0.200, SNI: 0.767 ± 0.160, Normal: 14.623 ± 0.824; shmTOR vs. shCON *n.s.*, shmTOR vs. SNI *n.s.*, shmTOR vs. Normal *p* < 0.001, shCON vs. SNI *n.s.*, shCON vs. Normal *p* < 0.001, SNI vs. Normal *p* < 0.001). However, on PID 7, the shmTOR group was significantly different from the shCON, SNI, and Normal groups. It showed that the treatment effectively alleviated pain hypersensitivity while both the shCON and SNI groups continued to exhibit hypersensitivity. No significant differences were found between the shCON and SNI groups, while those groups were statistically different from the Normal group (shmTOR: 5.979 ± 0.709, shCON: 1.954 ± 0.370, SNI: 0.919 ± 0.290, Normal: 13.163 ± 0.380: shmTOR vs. shCON *p* < 0.001, shmTOR vs. SNI *p* < 0.001, shmTOR vs. Normal *p* < 0.001, shCON vs. SNI *n.s.*, shCON vs. Normal *p* < 0.001, SNI vs. Normal *p* < 0.001). In the shmTOR group, the mechanical withdrawal threshold tended to gradually increase, reaching a peak on PID 21, whereas the shCON and SNI groups remained hypersensitive and the Normal group was maintained throughout the experiment (shmTOR: 8.773 ± 1.024, shCON: 2.282 ± 0.369; SNI: 2.442 ± 0.748, Normal: 13.840 ± 1.200, shmTOR vs. shCON *p* < 0.001, shmTOR vs. SNI *p* < 0.001, shmTOR vs. Normal *p* < 0.001, shCON vs. SNI *n.s.*, shCON vs. Normal *p* < 0.001, SNI vs. Normal *p* < 0.001) (Figure 2a,b). The Normal group showed no signs of mechanical hypersensitivity.

### 2.2. Activated mTOR Signaling Pathway Following the SNI and Its Downregulation Effects with rAAV-shmTOR Treatment

**Figure 3:**
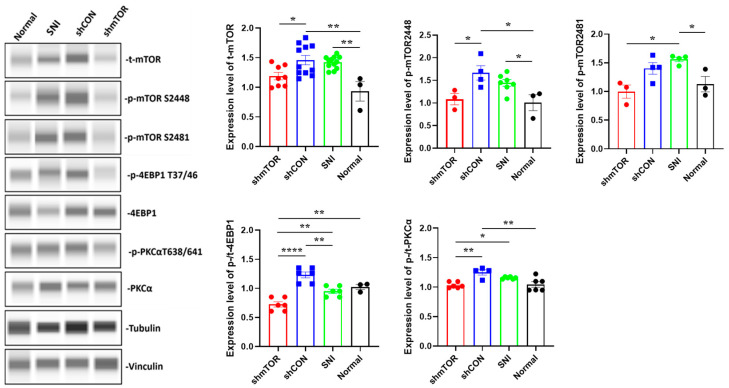
Western blot analysis for expression levels of mTOR and its downstream factors in the DRG on PID 21. Phosphorylated expressions of mTOR complex 1 and 2 were decreased in the shmTOR group, and its downstream effectors were downregulated as well. Data are expressed as mean ± SEM, * *p* < 0.05, ** *p* < 0.01, **** *p* < 0.0001. One-way ANOVA with Tukey’s multiple comparisons test.

To confirm that the mTOR signaling pathway is activated following injury, Western blot analysis was performed to evaluate the expression levels of total mTOR (t-mTOR), phosphorylated mTORC1 (p-mTOR 2448), mTORC2 (p-mTOR 2481), and the downstream effectors. The results indicated that SNI resulted in an increased t-mTOR expression compared to the Normal group, and the administration of rAAV effectively reduced t-mTOR expression, with no statistically significant differences between the shmTOR and Normal groups (shmTOR: 1.188 ± 0.062, shCON: 1.459 ± 0.078, SNI: 1.419 ± 0.03, Normal: 0.932 ± 0.164; shmTOR vs. shCON *p* < 0.05, SNI vs. Normal *p* < 0.01, shCON vs. Normal *p* < 0.01, shmTOR vs. Normal *n.s.*). Consistent with the behavioral test data, it has shown that the injury significantly increased the expression levels of p-mTORC1 (S2448) and p-mTORC2 (S2481) compared to the Normal group, while they were significantly downregulated in the shmTOR group on PID 21. The differences in p-mTOR S2448 expression levels in the shmTOR group were statistically significant when compared with the shCON group (shmTOR: 1.085 ± 0.126, shCON: 1.665 ± 0.159, SNI: 1.443 ± 0.727, Normal: 1.008 ± 0.175; shmTOR vs. shCON *p* < 0.05, SNI vs. Normal *p* < 0.05, shCON vs. Normal *p* < 0.05, shmTOR vs. Normal *n.s.*). For p-mTOR S2481, shmTOR group was only significantly different to SNI group (shmTOR: 1.0 ± 0.115, shCON: 1.403 ± 0.101, SNI: 1.560 ± 0.039, Normal: 1.128 ± 0.140; shmTOR vs. shCON *n.s.*, SNI vs. Normal *p* < 0.05, shmTOR vs. SNI *p* < 0.05, shmTOR vs. Normal *n.s.*, shCON vs. SNI *n.s.*, shCON vs. Normal *n.s.*), but it still showed some signs of decrease in the shmTOR group. Notably, to further investigate the feedback signal from the mTOR downstream effectors, we also evaluated changes in p/t-4EBP1 and p/t-PKCα, which reflect the activation of mTORC1 and mTORC2, respectively. The expression levels of p/t-4EBP1 in the shmTOR group were significantly lower than in the other groups (shmTOR: 0.724 ± 0.045, shCON: 1.232 ± 0.051, SNI: 0.950 ± 0.036, Normal: 1.024 ± 0.044; shmTOR vs. shCON *p* < 0.0001, SNI vs. Normal n.s., shCON vs. Normal n.s., shmTOR vs. Normal *p* < 0.01, shmTOR vs. SNI *p* < 0.01, shCON vs. SNI *p* < 0.01). It was also found that the p/t-PKCα expression level significantly decreased in the shmTOR group (shmTOR: 1.032 ± 0.021, shCON: 1.249 ± 0.045, SNI: 1.156 ± 0.01, Normal: 1.045 ± 0.05; shmTOR vs. shCON *p* < 0.01, SNI vs. Normal n.s., shCON vs. Normal *p* < 0.01, shmTOR vs. Normal n.s., shmTOR vs. SNI *p* < 0.05) (Figure 3).

### 2.3. Decreased Glial Cell Activation and Phosphorylated p38 (p-p38) in the DRG with rAAV-shmTOR Treatment

**Figure 4:**
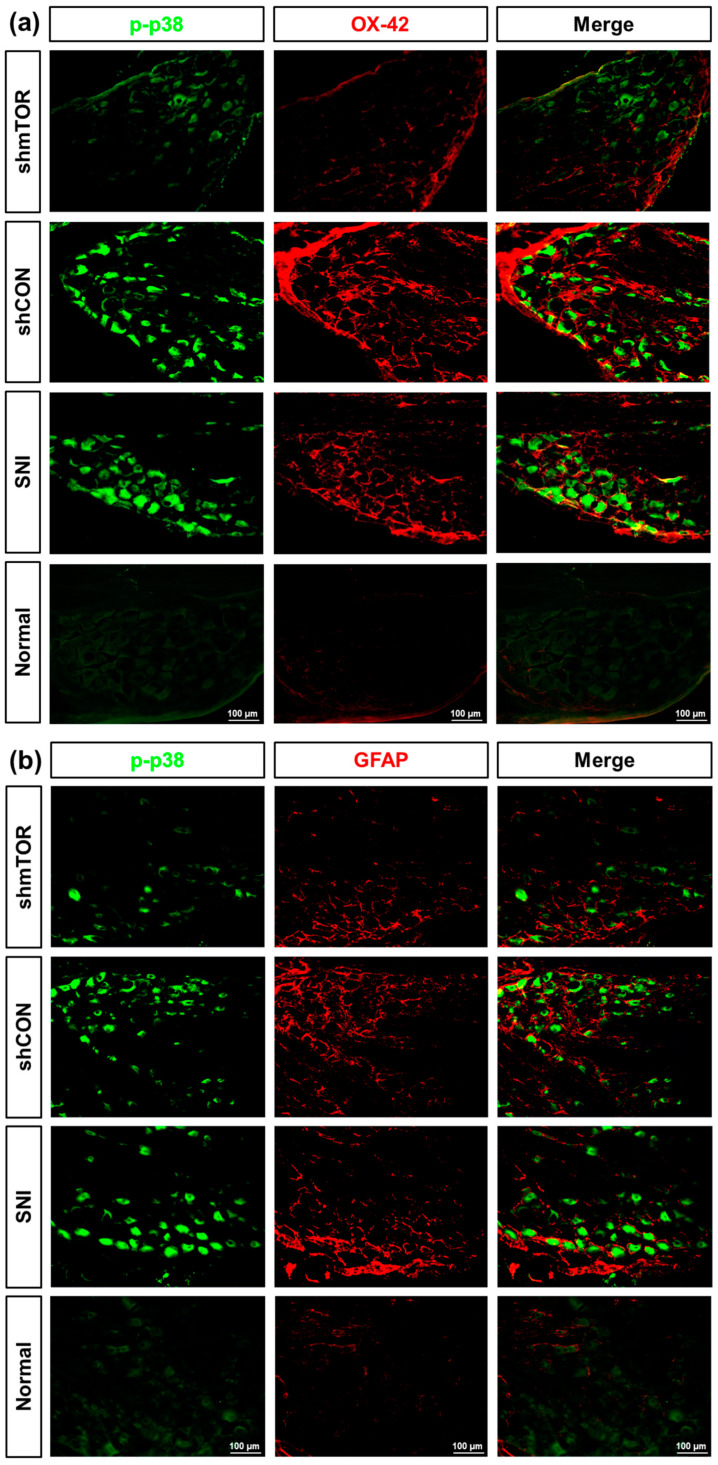

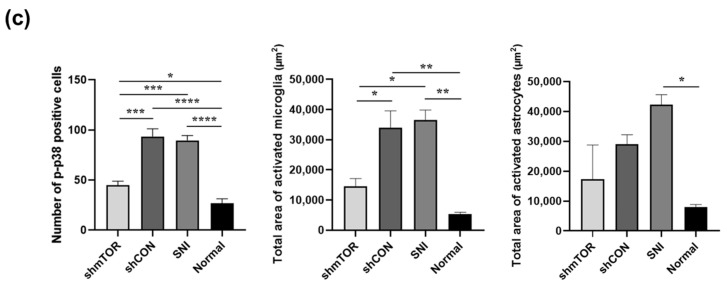
Immunohistochemical staining for p-p38, microglia, and astrocyte expression in the DRG on PID 21. Scale bar = 100 µm. (**a**) Expression of p-p38 and microglia. Increased p-p38 and microglia expressions in the SNI and shCON groups, while their downregulated expression in the shmTOR group. (**b**) Expression of p-p38 and astrocytes. No statistical significance in the shmTOR and shCON groups. (**c**) Quantitative analysis on p-p38 and activation of glial cells. Data are expressed as mean ± SEM, * *p* < 0.05, ** *p* < 0.01, *** *p* < 0.001, **** p < 0.0001. One-way ANOVA with Tukey’s multiple comparisons test.

To examine whether prolonged glial activation is a key contributor underlying the mechanism of chronic pain, we examined whether the inhibition of the mTOR signaling pathway reduced glial cell activation and p-p38 expression, which play a critical role in glial activity. It was found that nerve injury persistently activated glial cells and p-p38, which was predominantly colocalized with the neuronal marker in the DRG on PID21, but not with those that express CGRP (Figure S1a,b). As shown in Figure 4, SNI significantly increased the expression level of p-p38, OX-42 (microglia marker), and GFAP (astrocyte marker), while p-p38-positive cells and microglial activation were significantly decreased in the shmTOR group relative to the higher expression level of nerve-injured groups. Astrocyte activation showed a decreasing pattern in the shmTOR group, but it was only statistically significant between SNI and Normal groups. No statistically significant differences were found between other groups (p-p38, shmTOR (*n* = 4): 44.99 ± 3.979, shCON (*n* = 4): 93.60 ± 7.522, SNI (*n* = 4): 89.52 ± 4.941, Normal (*n* = 3): 26.76 ± 4.501, shmTOR vs. shCON *p* < 0.001, shmTOR vs. SNI *p* < 0.001, shmTOR vs. Normal *n.s.*, shCON vs. SNI *n.s.*, shCON vs. Normal *p* < 0.001, SNI vs. Normal *p* < 0.001; OX-42, shmTOR (*n* = 3): 14,608 ± 2535, shCON (*n* = 4): 34,005 ± 5581, SNI (*n* = 4): 36,555 ± 3295, Normal (*n* = 3): 5294 ± 694.8, shmTOR vs. shCON *p* < 0.05, shmTOR vs. SNI *p* < 0.05, shmTOR vs. Normal *n.s.*, shCON vs. SNI *n.s.*, shCON vs. Normal *p* < 0.01, SNI vs. Normal *p* < 0.01; GFAP, shmTOR (*n* = 4): 17,289 ± 11,469, shCON (*n* = 3): 29,071 ± 3121, SNI (*n* = 4): 42,282 ± 3322, Normal (*n* = 4): 7965 ± 872.4, SNI vs. Normal *p* < 0.05) (Figure 4a–c).

## 3. Discussion

In this study, we highlighted the role of the mTOR signaling pathway in a neuropathic pain model and suggested its association with p38 mitogen-activated protein kinase (MAPK) pathway in response to rAAV-shmTOR treatment. We observed a significant reduction in pain hypersensitivity, and a single treatment with a low dose of rAAV produced long-term efficacy that lasted over three weeks after the injury. To demonstrate the downregulation of the hyperactivated mTOR, we observed that the treatment decreased both p-mTORC1 and p-mTORC2, ultimately leading to the downregulation of the downstream effectors p-4EBP1 and PKCα. In addition, it was found that nerve injury significantly caused the persistent activation of p-p38 in neurons, but not in those that express CGRP, and glial cells on PID 21. This study suggested that hyperactivated mTOR in the DRG is clearly implicated in chronic pain model and can be reversed by a single injection of rAAV-shmTOR.

We chose rAAV-shmTOR as an mTOR inhibitor because it can downregulate both mTORC1 and mTORC2, preventing the incomplete suppression of hyperactivated mTOR. Although different animal models and administration routes have been widely used, it is interesting to note that our results are distinct from those of other mTOR inhibitor studies in terms of the treatment timepoint. In most previous studies, treatments were injected daily, several hours apart, or 1, 3, and 7 days after neuropathic surgery, and neuropathic models were established in a relatively short time [22,23,24,25]. In addition, despite the repeated and high volume of mTOR inhibitor treatments, only a few lasted longer than two weeks, at best. In contrast, in our experiment, we used a fully developed model of chronic pain, waited until post-operative day (POD) 16, and injected rAAV-shmTOR on that day, which most closely resembles actual chronic pain patients. Our data suggested that dual inhibition of the complexes has been shown to be more effective in prolonging treatment effects in chronic pain. Notably, given the fact that a high dose of AAV injection, such as 3 × 10^12^ vg/kg intravenously or intrathecally, could cause DRG toxicity [26,27,28], it is noteworthy that we injected a low dose of rAAV (1.5 × 10^8^ vg/200 g) even with a single injection. Although we did not perform a specific test for AAV toxicity in this study, no suspicious pathological phenotypes were observed in the histological results. In further study, ganglionopathy or axonopathy should be examined in a dose-dependent manner, an issue that is becoming increasingly significant in gene therapy. 

Numerous studies have demonstrated molecular interactions between spinal dorsal horn neurons and glial cells in neuropathic pain [29,30,31]. However, research on the cellular interactions of DRG sensory neurons is relatively limited. The MAPK family (ERK, p38, and JNK) transduces extracellular stimuli into intracellular responses through transcriptional or non-transcriptional regulation. Of the MAPK family, p38 is activated by cellular stress and cytokines and is known to regulate the synthesis of inflammatory mediators [32,33,34]. Yu et al. found that macrophages in the DRG are the primary contributors to the initiation and maintenance of chronic pain because macrophage-depleting treatment in the DRG did not decrease microglial numbers in the spinal cord with daily treatment [7]. In accordance with the findings of Yu et al., we also found that mechanical hypersensitivity and activated glial cells in the DRG were reversed by the shmTOR treatment, regardless of spinal gliosis (not presented). In the current study, we discovered an upregulated expression of p-p38 specifically in neurons, but not in those that express CGRP, and it has been previously reported that most mTOR-expressing sensory neuron fibers do not express CGRP [35]. Glial cells wrapping sensory neurons were not completely colocalized with p-p38-positive cells, and it can be inferred that a persistent glial activation may have been mediated by other cytotoxic factors or must be due to secondary effects, which is independent of p38 activation. The primary sensory neuron and satellite glial cells communicate through the release of various chemical messengers such as ATP, cytokines, and chemokines. It has been well established that an increased excitability in the DRG neurons following an injury plays a role in the structural and functional alterations of satellite glial cells. However, detailed mechanisms have not been elucidated yet. In further study, it would be important to understand their chemical communications to investigate alterations in proinflammatory mediators that may cause a persistent activation of glial cells. Furthermore, we observed pathological changes in the structure of the sciatic nerve after the injury. The SNI group exhibited atypically thin myelin sheaths and a reduced number of Schwann cells, whereas the shmTOR group displayed axons and myelination of relatively normal size. Nevertheless, in the shmTOR group, there were still instances of abnormal myelin infoldings that require further examination to quantify (Figure S2). 

Research on AAV therapy in rat models of neuropathic pain has the potential to offer valuable insights into potential treatments for human patients suffering from neuropathic pain. By studying the effects of AAV therapy in rats with neuropathic pain, researchers can comprehensively assess the safety and efficacy of this approach, optimizing parameters such as vector delivery, dosage, and targeted genes. These findings can help refine the development of AAV-based therapies for neuropathic pain in humans. In a rat model of neuropathic pain, there are also notable sex differences that have been observed. Research has shown that in some cases, male rats may display an increased sensitivity to neuropathic pain stimuli, while female rats may exhibit more variability in their pain responses, influenced by hormonal fluctuations during their estrous cycle. These sex-related distinctions are of significant importance in the development of potential treatments and therapies for neuropathic pain, as they could affect treatment efficacy and the underlying mechanisms of pain perception. Although this study did not specifically investigate sex differences, it is worth noting that exploring this aspect in future research could provide valuable insights for pain management to be more effective for both male and female patients with neuropathic pain.

## 4. Materials and Methods

### 4.1. Animals

Adult male Sprague Dawley rats (Orientbio, Pyung-tak, Korea) weighing 190–220 g were used in this study. Rats were divided into shmTOR (*n* = 29), shCON (*n* = 23), SNI (*n* = 13), and Normal (*n* = 8) groups. All animal experimental procedures were conducted in compliance with the Guide for the Care and Use of Laboratory Animals of the National Institutes of Health and approved by the Institutional Animal Care and Use Committee (IACUC:2022-0034) of Yonsei University. Animals were housed in groups of three in laboratory cages with food and water available ad libitum under a 12 h light/dark cycle (lights on at 07:00) in a room with controlled temperature (22 ± 2 °C) and humidity (55 ± 5%).

### 4.2. Spared Nerve Injury (SNI) Model

Under isoflurane anesthesia, the skin on the left lateral surface of the thigh was incised to expose three terminal branches of the sciatic nerve: the sural, common peroneal, and tibial nerves. After exposure, the common peroneal and tibial nerves were tightly ligated with 5.0 silk and sectioned distal to the ligation, removing 2–4 mm of the distal nerve stump. The sural nerve remained intact, and the muscle and skin were closed in two layers. For the SNI group, the animals went through the same procedure as above, but none of the viruses were injected.

### 4.3. Cell Culture

HeLa cells were obtained from the American Type Culture Collection (Manassas, VA, USA), cultured in Dulbecco’s modified Eagle medium (Thermo Fisher Scientific; Waltham, MA, USA) supplemented with 10% fetal bovine serum (Corning Inc., Corning, NY, USA), 2 mM GlutaMAX-1 (ThermoFisher Scientific), and penicillin (100 IU/mL)/streptomycin (50 μg/mL) (Corning), and incubated at 37 °C under a humidified 5% carbon dioxide (CO_2_).

### 4.4. Construction and Preparation of rAAV

The rAAV2 vector simultaneously expressed short-hairpin RNA (shRNA) and GFP. The shRNA was expressed under the control of the human H1 polymerase III promoter (pH1), while the opposite-orientation GFP expression cassette was under the control of a CMV promoter with a bovine growth hormone polyA signal (Figure 1b). Control shRNA sequences were provided by Dharmacon Inc. (Lafayette, CO, USA). The shRNA sequences against mTOR have been previously described [36]. All viruses were supplied by CdmoGen Co., Ltd. (Cheongju, Republic of Korea). Viral titers were determined using real-time qPCR, as described previously.

### 4.5. rAAV Injection

On post-operative day (POD) 16, the sciatic nerve proximal to the SNI lesion was carefully exposed. A 3 µL volume of a solution containing 1.5 × 10^8^ total particles of either rAAV-shmTOR-GFP or rAAV-shCON-GFP was injected into the upper sciatic nerve using a pulled glass capillary tube (HAVARD apparatus 30-0037) to ensure the safe delivery of AAV and minimize the damage to the sciatic nerve (Figure 1a). The capillary remained still for 3 min after the rAAV injection. After withdrawal, the muscles and skin were approximated and closed in layers using sutures. Rats were allowed to recover on a thermoregulated heating pad.

### 4.6. Behavioral Test

The von Frey test was used to test the mechanical withdrawal threshold, which was then quantified using the up–down method. A series of calibrated von Frey filaments (Stoelting, Wood Dale, IL, USA) was applied on the lateral surface of the left hind paw, and sudden paw withdrawal, flinching, and paw licking were counted as positive responses. To ensure that the injury-induced mechanical allodynia was rescued by AAV injection, a behavioral test was performed before modeling, on POD 16 and PID 3, 7, 14, and 21 (Figure 1a).

### 4.7. Immunohistochemistry

The animals were sacrificed on PID 21 and anesthetized intraperitoneally with a combination of ketamine (75 mg/kg) and xylazine (4 mg/kg). Perfusion was performed with 0.9% normal saline and 4% paraformaldehyde in 1× PBS. After perfusion, DRG L4-L5 were dissected with spinal cord, post-fixed for 2 h at 4 °C, and then transferred to a 30% sucrose solution overnight at 4 °C. DRG were sectioned into 15 µm thick sections using a Leica CM1850 cryostat and immediately mounted on coated microslides. A total of 18 sections per subject were used for each staining. Tissue sections were outlined using a PAP pen and allowed to dry completely at room temperature for 30 min. Sections were washed with phosphate-buffered saline with Tween 20 (PBS-T; PBS pH 7.4, 0.3% Triton X-100) three times for 5 min with gentle pipetting and vacuum aspiration, and incubated in a blocking solution (5% normal goat serum) with Triton X-100 (0.3%) for 1 h in a humidified box at room temperature. The sections were then incubated overnight at 4 °C with the following primary antibodies: OX-42 (MCA275G, 1:300), GFAP (ab7260, Abcam, 1:300, Cambridge, UK), phospho-p38 (9211, Cell Signaling 1:300), and GFP (A21311, Thermo Fisher Scientific; 1:300). After three washes with PBS, sections were incubated with secondary antibodies for 2 h. For GFP, the secondary antibody was removed and mounted after washing. The secondary antibodies included goat anti-rabbit (Alexa Fluor 488, A11008, 1:500, Invitrogen, Carlsbad, CA, USA) and goat anti-mouse (Alexa Fluor 594, A11005, 1:500, Invitrogen). The tissue sections were then washed, slide-mounted, and coverslipped with Vectashield Hardmount (Vector Laboratories, Malvern, PA, USA). Slides were observed under Axio Imager M2 (Carl Zeiss, Oberkochen, Germany) and using ZEN 2010 software v3.1 (Carl Zeiss, Oberkochen, Germany).

### 4.8. Western Blot Analysis

L4-L5 DRGs were quickly harvested and stored at −80 °C. DRGs were homogenized and lysed using the M-PER protein extraction reagent (#78501, Thermo Fisher Scientific). Total protein was quantified using a BCA protein assay (#23227, Thermo Fisher Scientific). The expression level of each target protein was analyzed using a capillary electrophoresis-based Western blotting system (Jess, ProteinSimple, Santa Clara, CA, USA), in accordance with the manufacturer’s protocols. Briefly, tissue lysates were combined with 5× fluorescent master mix to a concentration of 1 mg/mL and then heated at 95 °C for 5 min. Three microliters of sample were loaded into each well in the separation modules; protein separation, antibody incubations, washes, and detection were all performed automatically within the Jess, which utilizes a microfluidic capillary-based system and automatically analyzes protein expression levels by generating individual peak profiles for each protein. We used two types of separation modules depending on the molecular weight of the target protein (12–230 kDa module for 4EBP1, PKCα, and tubulin, and 66–440 kDa module for mTOR and vinculin). The raw data used in this study is based on the % area value of each peak provided by the Compass program, which was then divided by the value of vinculin or tubulin analyzed in the same capillary. From this, the relative values were analyzed based on the Normal group. Anti-mTOR (#2983, 1:100), anti-phospho-mTOR (S2448) (#2971, 1:50), anti-phospho-mTOR (S2481) (#2974, 1:50), anti-4EBP1 (#9644, 1:100), anti-phospho-4EBP1 (T37/T46) (#2855, 1:100), anti-PKCα (#2056, 1:50), and anti-phospho-PKCα (T638/641) (#9375, 1:20) were purchased from Cell Signaling Technology. Tubulin (#ab7291, Abcam, 1:200) and vinculin (#MAB6896, R&D Systems, 1:100) were used as the loading controls. The compass software v5.0.1 (ProteinSimple) was used to quantify the intensities of all protein bands.

### 4.9. Statistical Analysis

All data are expressed as the mean ± standard error of the mean. The statistical significance of differences between groups was calculated using a two-way or one-way analysis of variance followed by Tukey’s multiple test comparisons for behavioral analysis and Western blot analysis. Statistical significance was set at *p* < 0.05. All statistical analyses were performed using SPSS version 20 (SPSS Inc., Chicago, IL, USA) and GraphPad Prism 8 software (GraphPad Software Inc., San Diego, CA, USA).

## 5. Conclusions

This study contributes to our understanding of the role of the mTOR signaling pathway and the functional effects of p38 MAPK in a chronic pain model. We first attempted a long-term therapeutic strategy for neuropathic pain with a low dose of AAV injection by interfering with the mTOR signaling pathway, suggesting its potential application in pain treatment. Since mTOR signaling is broadly implicated in cancer, metabolic disease, and neurological diseases, dual inhibition of the mTOR signaling pathway through gene therapy may be the most promising clinical strategy beyond neuropathic pain.

## Data Availability

The data presented in this study are available on request from the corresponding author.

## Supplementary Materials

The following supporting information can be downloaded at: https://www.mdpi.com/article/10.3390/ijms242115918/s1.
