# Supplementary material for: A Single Injection of rAAV-shmTOR in Peripheral Nerve Persistently Attenuates Nerve Injury-Induced Mechanical Allodynia
Source: Int J Mol Sci. 2023 Nov 2;24(21):15918. doi: 10.3390/ijms242115918 (PMC10649356; doi:10.3390/ijms242115918)

Supplementary Figures.

Figure S1. Colocalization of p-p38 and NeuN in nerve-injured groups. (a) Immunohistochemical staining of p-p38 and NeuN. (b)Immunohistochemical staining of p-p38 and CGRP. It was found that nerve injury-induced p-p38 activation occurred predominantly in neurons, but not colocalized with CGRP.

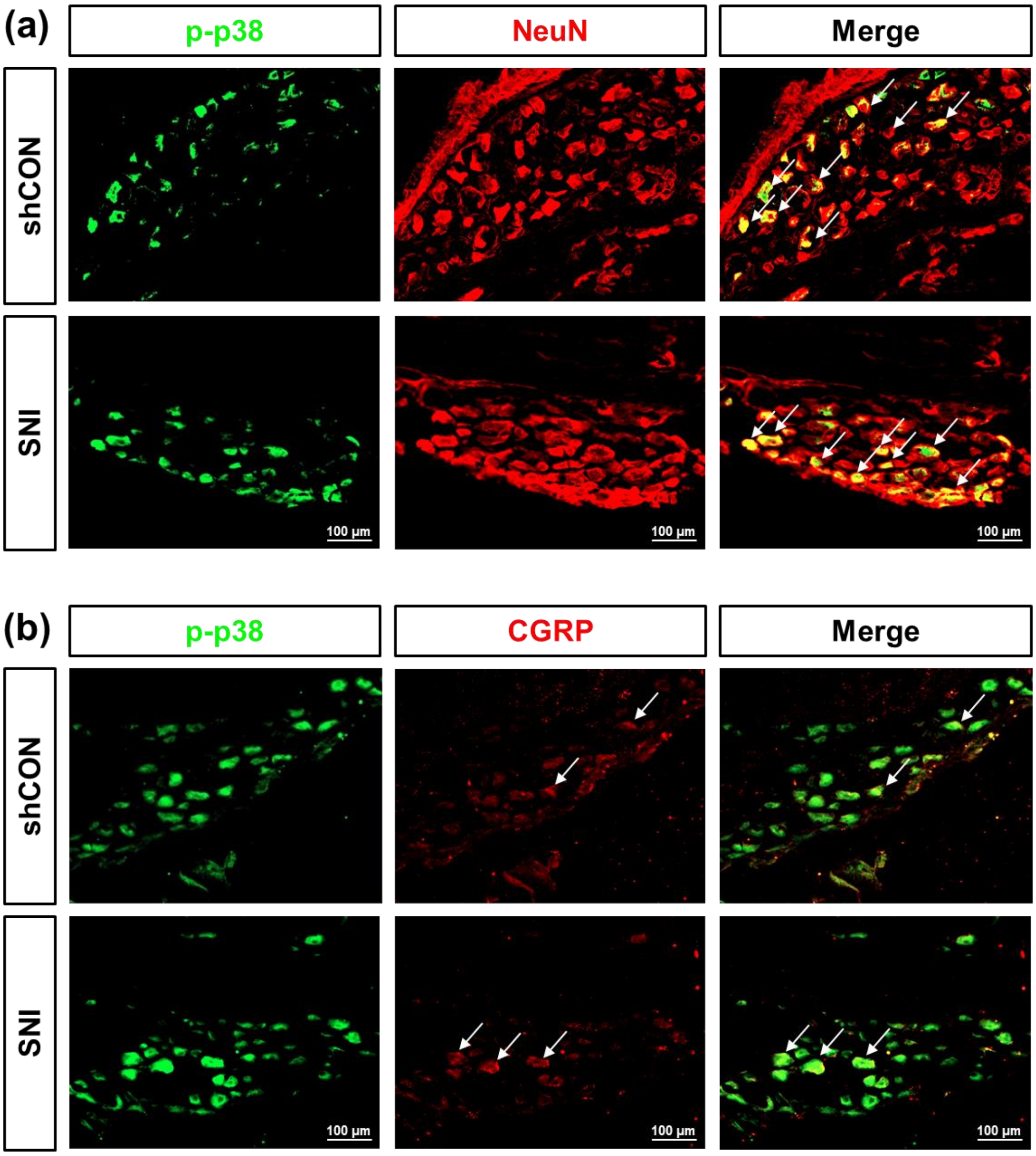

Figure S2. Ultrastructure microscopy of the sciatic nerve on PID21 using light microscopy. Ultrastructure microscopy of the sciatic nerve on PID 21 was conducted using light microscopy (Olympus upright microscope BX43). In the shCON and SNI group, the structure was remarkably less dense, and numerous pathological morphologies, hypermyelinated or abnormally thin axons were observed. The treatment group, on the other hand, had a relatively regular size of axons and myelin sheath, but it still shows some aberrant myelin infoldings.

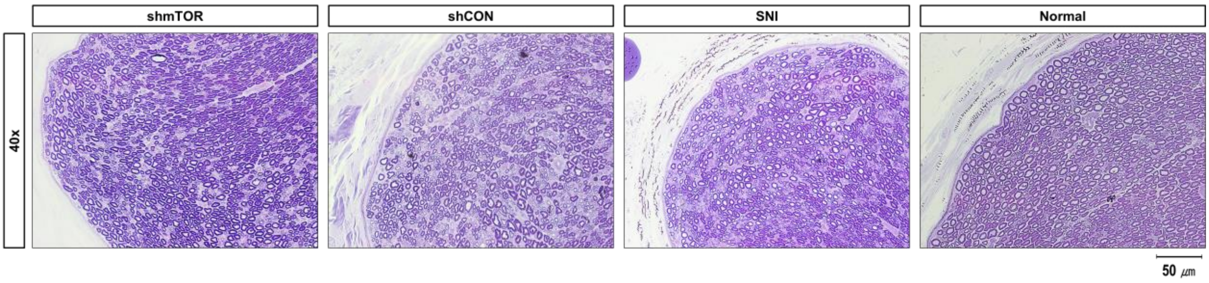

Supplement: Supplementary file 1 [file ijms-24-15918-s001.zip › ijms-2638060-supplementary.pdf]
